# Have we hit the JAK‐pot? Success of selective JAK 1 inhibitor following failure of pan‐JAK inhibitor in refractory pediatric ulcerative colitis

**DOI:** 10.1002/jpr3.70005

**Published:** 2025-02-10

**Authors:** Jessica A. Black, Brad Pasternak

**Affiliations:** ^1^ Phoenix Children's Hospital, Banner University Medical Center – Tucson University of Arizona Tucson Arizona USA; ^2^ Department of Gastroenterology Phoenix Children's Hospital – Phoenix Phoenix Arizona USA

**Keywords:** inflammatory bowel disease, Jak Kinase Inhibitor, tofacitinib, upadacitinib

## Abstract

The treatment options available for pediatric ulcerative colitis (UC) are challenging due to few with Federal Drug Administration approval. Newer medications approved for adults include additional biologics with differing mechanisms of action and small molecule drugs, such as Janus kinase (JAK) inhibitors. Our case outlines a patient with refractory UC who failed mesalamine, adalimumab, tofacitinib, and vedolizumab. She was evaluated for colectomy, but upadacitinib was trialed. She achieved rapid clinical remission and avoided surgery. This case demonstrates the failure of a pan‐JAK inhibitor (tofacitinib) and response to a more selective JAK 1 inhibitor (upadacitinib).

## 1 INTRODUCTION

Ulcerative colitis (UC) is a chronic inflammatory bowel disease (IBD) affecting the lining of the rectum and colon. Although medication options continue to develop, 10%–20% still require proctocolectomy.^1^ The only Federal Drug Administration‐approved agents for pediatric UC are infliximab and adalimumab, thus making changes when these fail to agents with different mechanisms of action more difficult. The newest agents approved in adults for IBD are the small molecule drugs, Janus kinase (JAK) inhibitors, tofacitinib, and upadacitinib. These have shown rapid onset of action and excellent efficacy and safety results in adult trials.^2^, ^3^ Data on its use in pediatric UC are limited. This case highlights a patient with inflammation refractory to numerous therapies, including tofacitinib, that ultimately responded to upadacitinib, thus indicating failure of one JAK inhibitor should not preclude consideration of another.

## 2 CASE REPORT

**Figure 1:**
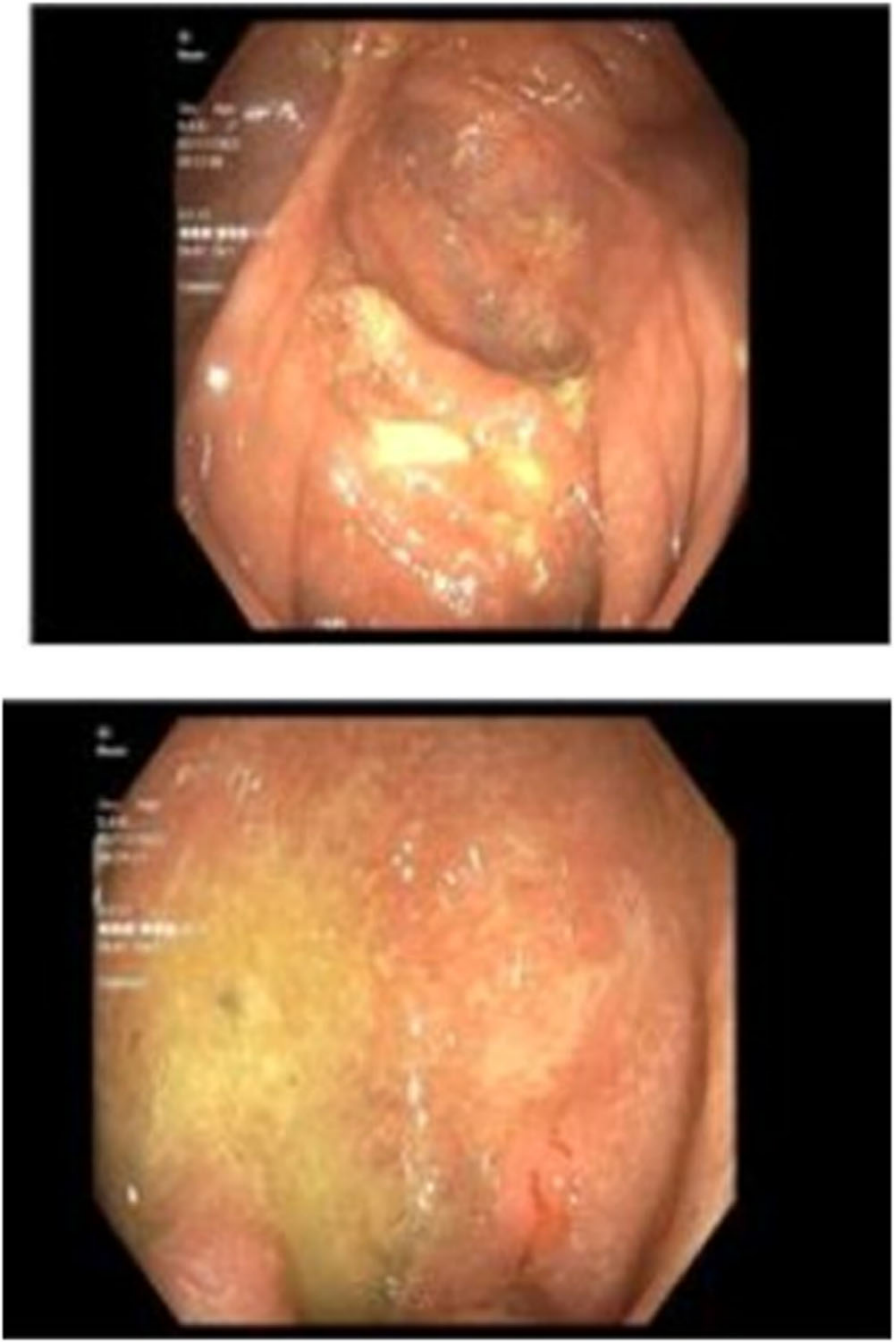
Erythema and granularity in the rectum and cecum (at diagnosis).

We present a 14‐year‐old female with refractory UC diagnosed in March 2022, initially with chronic active proctitis and a cecal patch with Paris classification E1 and a pediatric UC activity index (PUCAI) score of 40 (Figure 1). Weight at this time was 47.1 kg, with a body mass index of 19. Oral mesalamine 2.4 g daily, mesalamine 1000 mg daily suppository, and oral prednisone 40 mg daily were initiated with clinical improvement to PUCAI of 0. Four weeks after prednisone taper, she had worsening symptoms with a PUCAI of 25. Mesalamine dosing frequency was increased to 2.4 g oral twice daily, mesalamine 1000 mg daily suppository was continued, and oral prednisone was restarted at 20 mg twice daily. Hydrocortisone 100 mg daily enema was added. She initially improved but symptoms again returned when prednisone was discontinued with a PUCAI of 65. As a result, this regimen was discontinued, and she was admitted to the hospital for 1 week of intravenous (IV) methylprednisolone 40 mg daily and induction of adalimumab at 160 mg subcutaneous (SC) followed by 80 mg at Day 15. Her fecal calprotectin upon admission was >3000 mcg/g. Stool polymerase chain reaction was negative as well as *Clostridium difficile* toxin. Biochemical labs were unremarkable with hemoglobin of 12.5 g/dL, hematocrit of 37%, albumin of 3.8 g/dL, C‐reactive protein (CRP) < 0.4 mg/L, and erythrocyte sedimentation rate (ESR) of 12 mm/h. Her symptoms improved, and she was discharged on adalimumab 80 mg SC every other week. No oral steroid taper was indicated at this time. Adalimumab levels on follow‐up were 25, and no antibodies were detected.

**Figure 2:**
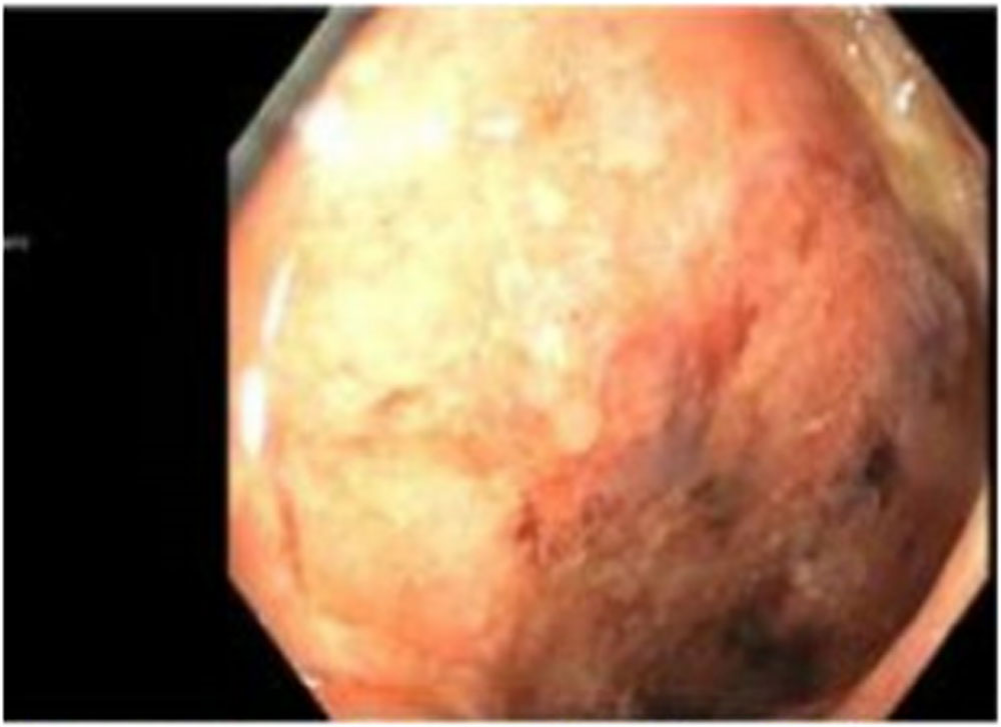
Severe left‐sided colitis on flexible sigmoidoscopy (at hospitalization).

One month following, her symptoms returned, and she was readmitted for IV methylprednisolone 40 mg daily. Laboratory revealed a fecal calprotectin 2310 mcg/g, ESR 29 mm/h, CRP 5.4 mg/L, and negative cytomegalovirus. Colonoscopy biopsies were significant for chronic severe active pancolitis with Paris classification E4 (Figure 2). Oral tofacitinib 10 mg twice daily was started with mild clinical improvement. A repeat flexible sigmoidoscopy showed significant disease progression in the rectum and sigmoid colon, thus pediatric surgery was consulted. The family opted to continue medical management. As a result, tofacitinib was discontinued with plan to utilize tacrolimus as a bridge to vedolizumab.

She began oral tacrolimus 4 mg twice daily with improvement seen over a week and a therapeutic level of 7.9 ng/mL. Vedolizumab 300 mg infusion was initiated, and tacrolimus 4 mg twice daily was continued upon discharge. Laboratory results after starting this regimen were fecal calprotectin 1650 mg/L, ESR 34 mm/h, and CRP < 3 mg/L. At follow‐up a few weeks later, she was in remission with PUCAI score of 5. She continued tacrolimus and vedolizumab at standard maintenance dosing for 6 months, with tacrolimus levels maintained between 6.5 and 17.9 ng/mL, ESR ranging from 7 to 34 mm/h, and CRP from <3 to 10.2 mg/L. Due to the risks of prolonged tacrolimus use, it was weaned at this time.

Soon after this wean, she developed a subsequent flare, thus it was restarted at the same dose. Another attempt of tacrolimus wean 3 months later was unsuccessful with a PUCAI of 20 and CRP 10.2 mg/L. She was again restarted on tacrolimus at the same dose with subsequent clinical remission and PUCAI of 0. Due to long‐term risk of tacrolimus, and need for monitoring, decision was made to switch to upadacitinib monotherapy.

**Figure 3:**
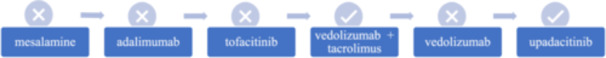
Overview of patient's treatment course.

Two months following the switch from tacrolimus and vedolizumab to monotherapy with upadacitinib 45 mg daily, her PUCAI score remained 0 with CRP <3 mg/L. She continues to remain in remission at the time of this manuscript preparation. She has had no adverse reactions. An overview of her treatment course can be seen in Figure 3.

## 3 DISCUSSION

First‐line therapy for mild/moderate UC is an aminosalicylate, both oral and rectal administration. This treatment inhibits the release of interleukin‐1, a major inflammatory molecule, and prevents leukocyte recruitment to the bowel wall to reduce inflammation in the lining of the intestine.^4^ When this fails, biologic therapy is typically initiated.^5^ Biologics are antibodies derived to target a specific mediator in the immune pathway. Biologics in UC target tumor necrosis factor (TNF), integrin, or interleukin 12/23, which all play a critical role in inflammation.

Once anti‐TNF fails, adult gastroenterologists have found success with small‐molecule drugs targeting JAK molecules. The four JAK molecules, JAK 1, JAK 2, JAK 3, and nonreceptor tyrosine‐protein kinase 2, are intracellular tyrosine kinases that activate immune pathways through a wide range of factors, including interleukins, interferons, and other molecules which lead to the inflammatory response in IBDs.^2^ After our patient failed mesalamine and anti‐TNF therapy, the small‐molecule tofacitinib was tried. This agent is a pan‐JAK inhibitor with preference for JAK 1 and JAK 2, which has shown promising results in adults. Following the failure of tofacitinib, tacrolimus, an immunosuppressant, was initiated as a bridge to vedolizumab, a biologic targeting integrin. There is data from Bousvarus et al. supporting this approach, which showed improvement in patients with corticosteroid‐resistant severe colitis, specifically allowing 93% of patients to be discharged without requiring surgery.^6^ While the combination of vedolizumab and tacrolimus was effective, each time tacrolimus was weaned, there was a relapse, suggesting the active agent maintaining her disease was tacrolimus. Although this agent is effective and safe short term, the long‐term efficacy is poor with a significant side effect profile.^7^ Serious risks include electrolyte derangements, renal failure, and malignancy, requiring very close monitoring. Ultimately, upadacitinib was started, and tacrolimus was discontinued.

Upadacitinib is a small‐molecule drug that selectively inhibits JAK 1. JAK 1 specifically is known to be required in the pathway of multiple pro‐inflammatory ILs, including IL‐6, IL‐7, IL‐9, IL‐15, and more.^8^ It is interesting that our patient failed tofacitinib, which is a pan‐JAK inhibitor, but responded well to upadacitinib, a more selective JAK 1 inhibitor. This suggests that patients may respond differently to different JAK inhibitors, and more selective inhibitors may be more efficacious. Peyrin‐Biroulet et al. published a systematic review and meta‐analysis in 2022 comparing biologics and small molecule drugs in treatment of moderate to severe UC in adults and found upadacitinib to be significantly superior in the induction of clinical remission compared to others studied, including tofacitinib.^9^

Once patients with severe UC fail corticosteroids and biologics, colectomy is often strongly considered. Our patient was evaluated for proctocolectomy before trying vedolizumab and small molecules. The family was insistent on trying all medical options, and she continues to be in remission on upadacitinib. This study demonstrates the importance of re‐evaluating the algorithm for acute severe UC and considering small‐molecule drugs before resorting to proctocolectomy.

## CONFLICT OF INTEREST STATEMENT

The authors declare no conflicts of interest.

## ETHICS STATEMENT

Informed patient consent was obtained from the family for publication of this manuscript.
